# Multidimensional Integrative Genomics Approaches to Dissecting Cardiovascular Disease

**DOI:** 10.3389/fcvm.2017.00008

**Published:** 2017-02-27

**Authors:** Douglas Arneson, Le Shu, Brandon Tsai, Rio Barrere-Cain, Christine Sun, Xia Yang

**Affiliations:** ^1^Department of Integrative Biology and Physiology, University of California Los Angeles, Los Angeles, CA, USA; ^2^Bioinformatics Interdepartmental Program, University of California Los Angeles, Los Angeles, CA, USA; ^3^Molecular, Cellular, and Integrative Physiology Interdepartmental Program, University of California Los Angeles, Los Angeles, CA, USA; ^4^Institute for Quantitative and Computational Biosciences, University of California Los Angeles, Los Angeles, CA, USA; ^5^Molecular Biology Institute, University of California Los Angeles, Los Angeles, CA, USA

**Keywords:** multidimensional omics integration, integrative genomics, cardiovascular disease, genomics, transcriptomics, epigenomics, metabolomics, proteomics

## Abstract

Elucidating the mechanisms of complex diseases such as cardiovascular disease (CVD) remains a significant challenge due to multidimensional alterations at molecular, cellular, tissue, and organ levels. To better understand CVD and offer insights into the underlying mechanisms and potential therapeutic strategies, data from multiple omics types (genomics, epigenomics, transcriptomics, metabolomics, proteomics, microbiomics) from both humans and model organisms have become available. However, individual omics data types capture only a fraction of the molecular mechanisms. To address this challenge, there have been numerous efforts to develop integrative genomics methods that can leverage multidimensional information from diverse data types to derive comprehensive molecular insights. In this review, we summarize recent methodological advances in multidimensional omics integration, exemplify their applications in cardiovascular research, and pinpoint challenges and future directions in this incipient field.

## Introduction

Cardiovascular disease (CVD) is a highly prevalent complex disease involving multiple risk factors, pathological changes in diverse cell types, tissues, and organs, and multidimensional molecular perturbations. Common forms of CVD including coronary artery disease (CAD), myocardial infarction, and stroke are among the leading causes of death in the world and therefore demand a better understanding of the etiology. Thanks to the rapid advances of omics technology, we are experiencing an explosion of biomedical data that have the promise to improve our understanding of the molecular underpinnings of clinical phenotypes (1). Accompanying the growing data volume are bioinformatics methodologies and tools to analyze individual data types, as recently reviewed by us and others (2–4).

However, it is increasingly recognized that focusing on any particular type of data only offers limited insights into the mechanistic black box bridging molecular traits and disease phenotypes (5). This is due to the fact that biological processes do not operate through any isolated molecular data type but manifest collectively as molecular cascades and interactions across omics domains to affect CVD etiology. Only comprehensive integration of multidimensional omics data can effectively capture a holistic view of pathogenic mechanisms.

Through recent efforts directly addressing this critical need, a number of integrative genomics approaches have been developed to model the interplays of data from multiple omics domains in a step-wise or meta-analytical fashion (6–8). The mathematical foundations of various integrative methods (9) and the principles and applications of such methods in cancer-related domains (10) have been previously reviewed. These methodological advances have significantly improved our ability to leverage the available rich data to recapitulate the flow of regulatory signals from the genetic background to the eventual disease outcome. Multidimensional analysis also has the built-in advantage of filtering away noise through the aggregation of biological information from independent and diverse sources. Pioneering efforts applying multidimensional data integration have led to numerous novel discoveries of biomarkers, disease pathways, and potential therapeutic targets for CVD (4, 11–15).

In this article, we focus primarily on multidimensional integrative methods applicable to CVD. We first provide an overview of the basic data types and principles of multidimensional data integration and then summarize methodologies and tools along with their representative applications in CVD. Lastly, we summarize the remaining challenges in the field and point to future research directions to improve the effectiveness of multidimensional data integration.

## Omics Data Types and Biological Relationships Between Data Types

**Figure 1:**
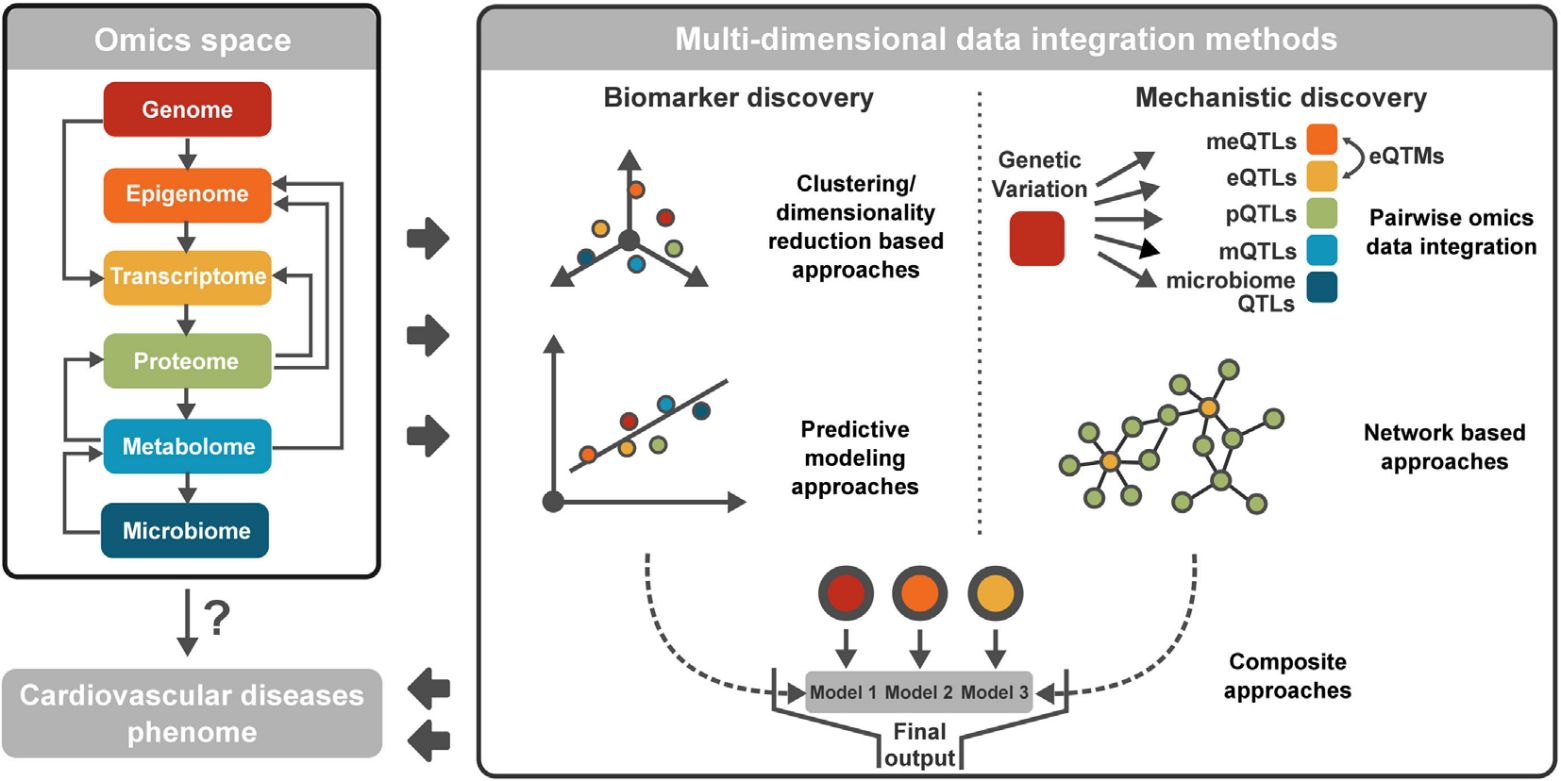
**Summary of different omics data types and multidimensional data integration methods**. Cardiovascular disease (CVD) involves various omics spaces and complex inter-omics interactions. To discover accurate biomarkers and disentangle disease mechanisms of CVD, multidimensional data integration methods are available, broadly categorized into clustering/dimensionality reduction-based approaches, predictive modeling approaches, pairwise omics data integration, network-based approaches, and composite approaches integrating multiple modeling approaches.

The most common omics data types representing the various molecular domains are genomics, epigenomics, transcriptomics, metabolomics, proteomics, and microbiomics (Figure 1). We have recently thoroughly reviewed the basic principles, the commonly used bioinformatics methods to analyze each data type, and their applications in CVD research (16). Briefly, genomics assesses DNA sequence and structural variations including single-nucleotide polymorphisms, insertions and deletions, copy number variations, and inversions. Epigenomics is the measurement of DNA methylation, histone modifications (methylation, acetylation, phosphorylation, DP-ribosylation, and ubiquitination), and non-coding RNAs (microRNAs, long non-coding RNAs, small interfering RNAs) (17). Transcriptomics evaluates the transcriptional activities of all genes, including the expression levels of individual genes and transcripts, as well as alternative splicing. Metabolomics aims to profile the levels and flux of metabolites. Proteomics captures the protein levels as well as post-transcriptional modifications of proteins. Lastly, microbiomics measures the composition of bacterial communities as well as the genome and transcriptome of individual bacterial species. Between the omics dimensions, intrinsic biological relationships exist (Figure 1), as detailed in our previous reviews (16, 18). Briefly, genomic and epigenomic variations have the capacity to control or modulate the transcriptome and in turn affect the proteome. Metabolites are products of host proteome, or derived from the gut microbiota, and can modulate the epigenome to affect transcription and translation. Gut microbiota can affect the host immune system and metabolism, which are central to programming many aspects of host activities. These complex cascades and interactions are critical elements for consideration in multidimensional data integration.

## Multidimensional Data Integration Methodologies and Example Applications in CVD

### Principles of Multidimensional Data Integration

Multidimensional data integration aims to aggregate information from diverse molecular domains into predictive models that can inform on mechanisms underlying pathogenesis or help select composite biomarkers that have diagnostic or prognostic values. A critical and non-trivial consideration for multi-omics integration is data preprocessing, including quality control and data normalization (19, 20). Proper preprocessing is important for removing outliers and non-biological variation within a data type and increasing the biological comparability between data types. To date, a vast majority of the recently implemented multidimensional data integration tools fall into one of the following five broad categories: clustering/dimensionality reduction-based methodologies, predictive modeling approaches, pairwise integration, network-based methodologies, and composite approaches, as summarized in Figure 1 and Table 1. The available methods are mostly designed for specific combinations of data types. The selection of proper methods requires consideration of data-driven statistical patterns and biological interpretability. However, depending on the specific applications, the weight for these two aspects may differ. Therefore, before choosing an appropriate method, it is imperative to first understand the biological question that is being addressed: biomarker discovery or mechanistic insight. For the discovery of diagnostic and prognostic biomarkers, data pattern is the key factor, whereas biological interpretation can be less important. Clustering/dimensionality reduction-based methodologies and predictive modeling methodologies are powerful for this task. For mechanistic studies, however, it is critical to couple intrinsic biological relationships among data types with data pattern searches to facilitate biological interpretation. Typically used methods here are pairwise integration and network-based approaches, although clustering/dimensionality reduction, predictive modeling, and composite methodologies can be used for both applications. In the sections below, we categorize and discuss the tools based on their general but not necessarily exclusive applications: biomarker or mechanism discovery.

**Table 1:** **Comparison of multidimensional data integration methodologies discussed in the manuscript**.

Method category	Brief description	Advantages	Limitations	Representative tools
Clustering/dimensionality reduction-based approaches	Transform data into common space through graph or kernel-based methods	Easy to implement using common statistical techniques; retain within-data properties; robust to different units of measurements and different data sets from the public domain	Cross-data interaction may be altered; application limited to visual overview of data and detection of subpopulations	Clustering-based: iCluster (21); ICM (22); TMD (23); SNF (24)
				Dimensionality reduction: Biofilter (25); CIA/MCIA (26); FALDA (27); GMDR (28)
Predictive modeling approaches	Machine learning based methodologies to predict prognosis or diagnosis and discover biomarkers	High predictive power; versatile methodologies; data-driven approach (does not require preexisting knowledge of omics interaction)	Overfitting issue; can require high computational power; does not integrate biological knowledge; higher accuracy requires larger data sets	Camelot (29); Kernel fusion (30); sMBPLS (31); MDI (32); PARADIGM (33); DIVIAN (34)
Pairwise omics data integration	Centered on interaction information between pairs of omics data	Easy to implement; reflects inter-omics interaction; causal implication	Available data dominated by expression quantitative trait loci (eQTLs); low robustness of *trans*-association signal	MERLIN (35); RAREMETAL (36); EMMA (37); GEMMA (38); PLINK (39); Matrix eQTL (40); SMR (41)
Network-based approaches	Reduce data complexity by converging multi-omics information onto networks	Networks can accommodate multiple layers of data; intuitive depiction and visualization of regulatory circuits	Computationally expensive; difficult to model feedback loops in multidimensional space	Weighted gene coexpression network analysis (42); MEGENA (43); Bayesian networks (44); TIGRESS (45); ARACNE (46); TIE* (47); GENIE3 (48); mixOmics (49)
Composite approaches	Flexible integration of multiple integration models	Flexibility and adaptability to diverse research needs	Few well-acknowledged frameworks available	Analysis Tool for Heritable and Environmental Network Associations (50, 51); Mergeomics (3, 52)

### Omics Integration Methodologies for Biomarker Discovery

#### Clustering/Dimensionality Reduction-Based Approaches

Clustering/dimensionality reduction-based approaches have the capacity to transform different data types into a common data space, thus facilitating downstream integration. This can be achieved through graph or kernel-based methods followed by grouping data features into a smaller number of variables. These approaches are the most straightforward methods to define biomarkers of disease or disease subtypes, thereby facilitating diagnosis and prognosis. The advantages of clustering/dimension reduction include the ability to retain within-data type properties and the robustness to different units of measurement. The drawback, however, is that the transformation of different data types may alter the underlying interaction between data types, even if within-data properties are retained (6).

Clustering-based approaches typically include hierarchical clustering (53), biclustering (54), and k-means clustering (21), which are used to find disease subpopulations (21, 55), refine disease characteristics, and help identify markers (56). Various methods such as iCluster (21), ICM (22), TMD (23), and others have been developed to use clustering for multidimensional integration (Table 1). For example, iCluster models the associations between different data types and the structure within each data type to bring the data onto the same feature space allowing for k-means clustering. This workflow has been applied on breast and lung cancer data sets to identify novel disease subtypes, which cannot be resolved using a single data type (21). We did not identify specific applications of multi-omics clustering in CVD research, although this type of approach has been applied based on individual data types (57–59). Future applications of such approach engaging multidimensional data will facilitate more accurate patient stratification based on multi-omics patterns and help identify unique biomarkers of CVD subtypes.

Dimensionality reduction can be achieved either intrinsically, which scales the dataset of interest using an analytical method, or extrinsically, which uses information outside of the dataset. Intrinsic approaches are the most widely used for dimensionality reduction of genomics data. Standard techniques include principle component analysis, factor analysis, multidimensional scaling, and others, which have been covered in a review of feature selection and extraction methods by Hira and Gillies (60). Tools utilizing dimensionality reduction techniques for multidimensional integration include CIA/MCIA (26), FALDA (27), and others (Table 1). Multifactorial dimensionality reduction has been applied by Badaruddoza et al. to identify environmental and genetic interactions in type 2 diabetes and CVD (61).

#### Predictive Modeling Approaches

Predictive modeling is another powerful data-driven approach that is primarily utilized for the discovery of composite biomarkers in a multi-omics, big data landscape. In broad terms, it comprises a set of algorithms capable of learning from data to make predictions, which theoretically become more accurate with increasing amount of data. A series of machine learning techniques are commonly implemented, including logistic regression, support vector machines, random forest, neural nets, Bayesian models, and boosting (62) to select the most predictive features. This is typically done through weighting, where the most predictive features contribute more weight to the final model.

Among the various predictive modeling approaches used for multidimensional data integration (Table 1), an example is Causal Modelling with Expression Linkage for cOmplex Traits (Camelot) (29). Camelot implements elastic net regression to select the most significant features and uses bootstrapping to reduce the set of features to potential causal genes (29). There has also been widespread usage of machine learning methods in CVD-related fields to identify CVD risk variants (34) and estimate cardiometabolic risks (63). Specifically, Chen et al. trained an ensemble classifier to prioritize non-coding risk variants using multi-omics data and found that the variants associated with repressed chromatin were often the most informative (34). Kupusinac et al. leveraged artificial neural networks to predict cardiometabolic risk using easy to obtain, non-invasive primary risk factors and achieved comparable performance to predictions based on more invasive secondary risk factors (63).

### Omics Integration Methodologies for Mechanistic Discovery

#### Pairwise Omics Data Integration

As discussed previously, there are intrinsic biological relationships between data dimensions that can inform on mechanisms, and quantitatively assessing the association between the omics domains can help capture such relationships in a data-driven manner. Pairwise omics data integration is therefore an intuitive and commonly used approach that characterizes interactions between two omics domains. This type of integration comes in two broad categories based on whether genetic information is under consideration (Figure 1). The first category is genetics of intermediate traits analysis, in which DNA variants are tested for association with downstream omics markers. The second category is correlation analysis between two non-genetic omics data types (e.g., between metabolites and microbiome).

For genetics of intermediate trait analysis, expression quantitative trait loci (eQTLs) are the most well-known pairwise integration where genetic variations are linked to transcriptomic alterations, achieved through an association test between variants and gene expression levels (64). There are numerous methods available to conduct eQTL analyses such as GEMMA (38) and Matrix eQTL (40), which have been discussed in detail elsewhere (65). Genetic loci can also be associated with omics data types other than transcriptomics, such as methylation quantitative trait loci (66), microRNA QTLs (miR-eQTLs) (67, 68), protein quantitative trait loci (69–71), metabolite quantitative trait loci (72–74), and microbiome quantitative trait loci (75). Correlations between downstream omics data are also informative, although it may be difficult to infer a causal relationship. For example, expression quantitative trait methylation has been defined as the correlation of CpG methylation levels to gene expression (66). This type of analysis can be extended to the other omics data types (e.g., between microbiome and metabolome).

The combination of genetics-based and non-genetic correlative analyses can help infer causality. This concept has been widely used in CVD research to infer candidate causal genes (12, 76–80). Schadt et al. (81) were among the first to develop a formal procedure to incorporate eQTLs, genetic disease association, and gene–trait correlation to infer disease causal genes. Yang et al. (76) applied this approach to identify tissue-specific causal genes for atherosclerotic lesions. Laurila et al. applied a combined approach using both eQTLs and pathway analysis to link genomics, adipose transcriptomics, and lipidomic profiling, highlighting a shift toward inflammatory HDLs in individuals with low HDL (82). Huan et al. (83) combined eQTLs, miRNA-eQTLs, correlative analysis between gene expression and microRNAs, and GWAS to identify microRNA–gene pairs that are putatively causal for CVD. In another effort toward this direction, Zhu et al. proposed a summary data-based Mendelian randomization method that integrates diverse types of QTLs with GWAS to infer candidate genes for complex traits (41).

#### Network-Based Approaches

Network approaches have emerged as another powerful platform for multidimensional data integration. Networks depict omics markers as nodes and connections between markers as edges that reflect correlations, regulatory relations, or physical interactions. There are many types of network inference approaches, including regression, mutual information, correlation, and Bayesian networks (44) (Table 1). Among the widely used network methodologies, particularly in the CVD field, are correlation-based methods such as the weighted gene coexpression network analysis (42). These approaches primarily focus on gene expression data and use correlation patterns to group functionally related genes into modules, which significantly reduce the complexity of overlaying other types of omics data onto transcriptomics. It is also feasible to apply these coexpression network approaches to other types of omics data (e.g., DNA methylation data).

In network-based applications, different data types are typically mapped to features (e.g., genes) that can be projected onto networks. For example, Huan et al. integrated coexpression networks with genetic variants to identify causal functional modules for coronary heart disease (78). Yao et al. built an eQTL coexpression network to reveal CVD-related modules (84). Shang et al. inferred a transcription factor regulatory network from blood macrophages transcriptomics profiles and identified a key driver, LIM domain binding 2, for atherogenesis (85). Public network depositories such as protein–protein interaction (86) and BioGRID (87) have also been used to identify novel candidate CVD genes from diverse datasets (88, 89). Recently, the Björkegren group integrated Bayesian networks with CAD genetics and transcriptomics data from CVD relevant tissue types and identified CVD-causal subnetworks and key drivers (80).

#### Composite Approaches

Many of the available tools and methods applied to better understand the etiology of a complex disease like CVD utilize combinations of the various principles discussed above (Table 1). The integration of the various methods and data types is typically done in a sequential manner where a common overlapping feature (e.g., genes) is used to convert the output of one part of the analysis to be a compatible input for the next step. One example is the Analysis Tool for Heritable and Environmental Network Associations (50, 51), which utilizes neural nets and has been previously applied to predict HDL cholesterol (90). Specifically, this method generates a separate neural net model for each individual data type, and the features with the top predictive power from each model are combined in an integrative model, which possesses higher predictive power than any of the individual models (91). An alternative approach is to employ a majority voting scheme from each of the independent models from the individual omics types, thereby avoiding the additional step of merging multiple models but still leveraging information from multiple data types to predict a clinical outcome (92). As another example, Inouye et al. constructed metabolic networks where metabolites were identified to be associated with the genes identified in the eQTL analysis, thereby layering an additional data modality. The expression levels of the prioritized candidate genes were found to be associated with the phenotypes of the disease, demonstrating the effectiveness of this integrative method (15). Our lab has recently developed a highly generalizable analytical framework, named Mergeomics (3, 52), to more effectively incorporate multidimensional data and various integration strategies. Mergeomics can reveal pathogenic processes underlying diseases by interrogating enrichment patterns from diverse omics association data, and then leverage tissue-specific networks to identify key perturbation points of the significant processes. With this approach, we have prioritized novel regulatory genes and therapeutic targets for CAD and hypertension from diverse genomics, transcriptomics, and molecular network resources (12, 79, 93).

## Challenges, GAPS, and Future Directions

The explosion of omics data in recent years has shifted the bottleneck of scientific discovery from data generation to the need for efficient multidimensional integrative methods. As summarized in this review, there have been major progresses in the development of methodologies and tools that can accommodate and integrate multidimensional data, and the application of these integrative approaches have yielded significant insights into the complex etiology of CVD. However, this field is still in its infancy, and the flexibility, effectiveness, and robustness of data integration to extract biological insights is still restricted. The limitations are mainly due to the intrinsic complexity within individual datasets and between datasets, as well as technical difficulties in integrative modeling that accurately captures true biological complexity. Moreover, there is currently no optimal tool with broad applicability in varying analytical scenarios, as most tools are tailored to particular applications and are limited in data type coverage, thus restricting their generalizability. Further, the performance of the various methodologies has not been comprehensively compared, and there is a lack of general guidance in the field on best practices. To address these challenges, future efforts should focus on intimate collaborations between computational biologists, systems biologists, and experimental biologists in the following areas. First, there is a need for a comprehensive map of data types and data relations, application scenarios, and the desired outcomes. Such a map will facilitate the design of flexible and generalizable multidimensional integration methods. For example, clear differentiation of diagnostic, mechanistic, and therapeutic needs will help choose more appropriate algorithms. Second, comprehensive testing and evaluation of various statistical and mathematical models and computational algorithms are needed to document the performance. The recent effort on network method comparison *via* crowd sourcing is one of the first demonstrations of the value of this approach (94). Performance evaluation should also go beyond *in silico* studies to engage bench scientists to systematically test predictions from the modeling studies to help refine the computational methods. With growing interests and coordinated efforts, multidimensional omics integration will be the next wave of modern biology to help dissect major complex diseases like CVD by promising a holistic understanding of disease pathogenesis and more accurate and personalized diagnostic and prognostic markers.

## Author Contributions

DA, LS, BT, RB-C, CS, and XY drafted and edited the manuscript.

## Conflict of Interest Statement

The authors declare that the research was conducted in the absence of any commercial or financial relationships that could be construed as a potential conflict of interest.
